# An ethnographic study of salt use and humoral concepts in a Latino farm worker community in California’s Central Valley

**DOI:** 10.1186/s13002-017-0140-4

**Published:** 2017-02-08

**Authors:** Judith C. Barker, Claudia Guerra, M. Judy Gonzalez-Vargas, Kristin S. Hoeft

**Affiliations:** 1 Department of Anthropology, History & Social Medicine and Center to Address Children’s Oral Health Disparities, University of California San Francisco, 3333 California Street, suite 485, San Francisco, CA 94143-0850 USA; 2 Helen Diller Family Comprehensive Cancer Center, Community Education & Outreach/Pasick Research Group, University of California San Francisco, San Francisco, CA USA; 3 Institute for Health Policy Studies, University of California San Francisco, San Francisco, CA USA; 4 Department of Pediatrics, Department of Preventive & Restorative Dental Sciences, and Center to Address Children’s Oral Health Disparities, University of California San Francisco, San Francisco, CA USA

**Keywords:** Salt, Humoral medicine, Migrant community, Rural, Latino, California’s Central Valley

## Abstract

**Background:**

This article reports on the use of domestic or table salt for its perceived health effects and healing properties in a Latino farmworker community. It explores how contemporary salt usage beliefs can be seen to have roots in long-standing humoral theories of medicine and health.

**Methods:**

This qualitative investigation comprised 30 in-depth individual interviews and five focus groups conducted in Spanish with Mexican and Central American immigrants in one small city in California’s Central Valley (*N* = 61 total participants). Interviews and focus groups were audiotaped, translated into English and transcribed. Several researchers independently and iteratively read transcripts, developed and applied codes, and engaged in thematic analysis.

**Results:**

Strongly emergent themes identified the importance of balance in health, and beliefs about the effects on salt on health. Valued for its culinary role, for bringing out the flavors in food, and used by people of all ages, salt use is part of a robust set of cultural practices. Salt was regularly mixed with foods in different combinations and ingested to restore balance, prevent disequilibrium or reduce vulnerability to diverse illnesses, promote rehydration, and address symptoms of exposure to extremes of temperature or physical or emotional stress. Statements made and practices engaged in by participants were highly suggestive of health and healing beliefs common to humoral belief systems based primarily on a hot-cold dichotomy in classifications of foods and healing behaviors. We evaluate these statements and practices in the context of the existing literature on historical and contemporary humoral beliefs in Latin American communities, in Mexico and Central America, and in the United States.

**Conclusion:**

Humoral theory is a useful framework for understanding contemporary rural Latino migrant farmworkers’ perceptions of the importance of salt for their health.

## Background

Humoral medicine and therapeutics, first codified in the ancient Greco-Roman system of thought, appear today in various forms in medical systems worldwide, including in Unani Tibb, Ayurveda and Traditional Chinese Medicine [1, 2]. For more than six decades there has been extensive discussion in the social, medical and ethno-pharmacological scientific literature on humoral medical concepts in relation to physical and mental disease and illness [3–8]. Major foci in that literature have been on the identification and use of plants and botanical products for therapeutic purposes and on the social and cultural rationales and contexts for their use, across history and in various cultural settings. A solid corpus of anthropological and ethno-botanical works features humoral medicine in Mexico and the Central and South American regions since pre-conquest times [8–17]. Relatively few reports exist, however, of how minerals or other naturally occurring non-vegetable substances are incorporated into humoral healing concepts or practices. Salt – domestic or table salt, also known as sodium chloride - is one non-botanical substance that has sporadically appeared in commentaries in the existing literature on humoral medicine in Latin America, but little work examines its integration into wider aspects of a humoral system of thought. This report, then, is among the first to feature salt as a main topic, and to focus on salt use in a Latino farmworker community in the United States.

We begin by presenting a truncated history of work on salt use and humoral pathology and therapeutics in pre-Conquest Mayan and Aztec times^1^^,^ 2 ^,^ 3, and then in more recent Latin American especially Mexican contexts from the mid-20th Century on. This background section is followed by a brief description of our study’s background, location and methods, and presentation of results. Beliefs and practices around domestic table salt and its perceived health effects and healing properties are explored in a Latino community in California’s Central Valley. We show contemporary salt use beliefs and practices are influenced by humoral beliefs that rural field and factory workers’ employ to manage occupational exposures, work endurance and health, particularly in conditions of unrelenting and uncontrollable daily stresses and extreme heat. Typical quotes illustrate and highlight the points made. Further discussion and interpretation of these results are made in relation to the existing body of literature on humoral medicine in the Americas. We conclude that many of the ideas and practices of humoral medicine are present in this contemporary Latino community and inform the material and symbolic use of salt as a culinary and therapeutic agent.

### Basic concepts of humoral medicine

The concept of humoral pathology, or humoral medicine, is widespread, with several major variants existing throughout Asia, Latin America, and Europe in both historical and contemporary times [1–6]. All variants are based on a model of balance between key material or metaphoric elements necessary for health, with disease or illness resulting from imbalance of these elements [1, 2]. Major European variants are based on the Hippocratic System, which has its origins in ancient Greco-Roman medicine. The Hippocratic System was based on the theory that all substances were made up of four elements – earth, air, fire, and water – in varying amounts. As a result substances possessed the qualities of two uni-dimensional concepts each comprised of two polar opposites: cold versus hot, and wet versus dry. This theory presented the idea that a healthy body is in a state of equilibrium or balance in each of these oppositions. Illness is equilibrium lost, oppositions out of balance: the body becomes too hot, too cold, too wet or too dry [2, 9, 10]. In the context of health, these properties do not refer to the physical temperature or wetness of these elements or substances, but rather to intrinsic qualities, metaphorical and symbolic, in substances such as food or medicines, and in particular environmental conditions [9, 10]. Various authors have examined the existence of similar, or complementary, concepts in the Americas pre-Conquest as well as the transmission of these concepts to Latin America post-Conquest, where the hot-cold dimension has become dominant and the other axes less prominent [9–20].

### Salt in pre-conquest Mesoamerican and Latin America

Throughout recorded history, sodium chloride (NaCl), commonly known today as table salt, has played an important role in Mesoamerican and Latin American culture, in both cooking practices and healing. Salt has been an essential component in the preparation and enjoyment of Mexican and Central American foods since Mayan and Aztec times [21, 22]. By the time the Spaniards arrived in the early sixteenth century, the Mayans had a vast salt industry and were trading in salt and salted (preserved) goods such as salted fish [23–25]. In addition to salt as a culinary item, it was a necessary part of daily life. “In the Maya area, for example, dietary salt needs were considerable because the tropical climate and hard work caused abundant salt loss through sweat. People who live in these conditions usually require a minimum of 8–10 g of salt a day” [24]. In ancient Mesoamerica, the Mayans also used salt, in combination with other elements, for other perceived health values; for example, as a form of birth control, in controlling epilepsy and in reducing the pain of childbirth [25].

For the Aztecs, after corn, chili peppers and salt were the most important food ingredients [24, 26]. The Aztecs believed in four young goddesses: sun, maize, fresh water, and salt (Huixtochiuatl, the Lady of Salt) [27]. Sahagún (cited in [26]) provides a rich description of the market of Tlatelolco in pre-Columbus times with vendors selling a variety of salt-cured fish and “squashes and chiles in varied colors from bright red and yellow to deep green… Most gorgeous of all were the edible flowers of cacao, squash, and maguey (Agave) plants… Nearby rose mountains of salt…” ([26], pp. 8–9). Chili peppers, which have been part of the Mexican diet for thousands of years ([28], p. 46), citrus fruit, introduced at the time of the conquest ([28], pp. 52–53), and salt for seasoning, used for millennia [24], are today frequently used to bring out flavors in Mexican food.

Like the Mayans, the Aztecs used salt as an element in healing. Salt was combined with concentrated maguey syrup (agave) to prevent infection in wounds, a practice later examined and claimed to be effective [29]. Historians suggest that the Aztecs had a view of causes and cures for disease that included supernatural (religious, magical) and natural (physical) elements ([18], p. 3). This duality in health beliefs was reflected in Aztec cosmology, which conceived of a universe of permanent conflict between oppositional forces; light and darkness, north and south, heat and cold [14, 18]. Perceived natural causes of illness (excesses, accidents, deficiencies, exposure to sudden temperature changes and contagions) were inextricably connected with the intervention of non-human beings with “more than normal power” [18].

### Contemporary humoral theory in the Americas: controversy and continuity

To better understand the long traditions, major continuities and controversies discernable in humoral medicine in Latin America, especially in relation to salt, we present next a summary of major concepts, beliefs and practices as described by seminal (mainly anthropological) authors since the 1950s. These works expand our knowledge of the concepts discussed thus far, and allow us to see how the fundamental theory of humoral medicine resulted in many apparently distinct local practices from a fairly unified set of principles. Understanding this variability underscores the value of understanding humoral concepts in migrant communities and demonstrates how migrants adapt, manipulate, mobilize, transport and meld these belief systems into practical schema. These practical schema also incorporate biomedical concepts and therapeutics, and so become mechanisms that provide literal as well as ‘symbolic’ relief from illness.

Although there were prior studies examining humoral beliefs in Latin America [30–34], anthropologist George M. Foster is often cited as the early major proponent of the idea that humoral beliefs found in Latin America can be traced back to ancient Greco-Roman humoral theory [9, 12, 14, 15, 35]. In a paper summarizing his 50 years of research on traditional medicine in the village of Tzintzuntzan, Michoacan in Mexico, Foster [9] states that upon the arrival of the Spanish conquistadors, different ways of interacting with the environment, foods and disease theories were introduced to the local populations including the Hippocratic humoral system. Among the Tzintzuntzan villagers, balancing hot and cold qualities could be naturally or materially manifest, but were mainly symbolic or metaphoric [9]. According to Foster, in humoral health practices decisions about treatments depended on various factors: symptoms, parts of the body affected, diagnosis of the cause of the illness, and prior experience with different treatments. He speaks of how illness caused by an “offending heat or cold may be neutralized or countered by mean of internal remedies” ([9], p. 812). Such remedies can be tea infusions, coffee and sometimes foods “of humoral qualities” that are opposite to the cause of the symptom [9].

Another well-known humoral scholar from that era was Madsen [10] who worked in San Francisco de Tecospa, a village at the southern end of the Valley of Mexico. The indigenous people of Tecospa also believed that the universe was ordered by a balance of opposites, two halves of one whole (sickness and health, war and peace, hot and cold). Regaining health was achieved by reestablishing the balance of the body - by consuming food and taking medicine with hot/cold properties perceived to be opposite to those producing sickness. Unlike Foster, however, Madsen argues that this “opposite” system was the same as the Aztec concept of the universe, and at least in part existed pre-Conquest. The Greco-Roman humoral concept of wet/dry was not found in Tecospa beliefs, but a system of ranking qualities by intensity was found in both pre-Conquest and the Hippocratic systems. A new category among the Tecospa had also been introduced: fresh and very fresh, which appeared to some degree to be in opposition to hot. Thus, food was classified as hot and very hot, cold and very cold, and fresh and very fresh [10]. The concept of “temperate” was also present, the result of balanced mixing of hot and cold. The mixing of cold and hot dishes, for example, creates temperate food. When in balance (i.e., when healthy), the human body is temperate [20]. Under the Tecospan system, chili and spicy foods are hot, whereas vegetables such as cucumbers and tomatoes are cold. Certain fruits are classified as fresh for the water that they contain (e.g., apples, grapes, grapefruit, lemon, lime and melon). The most important measure for determining the hot or cold classification of any food was its perceived effect upon the metaphoric temperature or balance of the body. Madsen observes that in situations of work in extreme heat in Tecospa, including work in the fields, fresh foods were perceived to be good because these help to return the body to its normal temperate state [10]. Similarly, in the hot and cold classification of the Zapotec people in Mitla, Oaxaca, Messer [11] reported that lemon is consistently classified as “very cold”, “cold” or “cool.” Taken internally or applied externally as part of a poultice, it was believed to “cool” overheated bodies, to relieve inflammation and to “cool” hot spicy dishes.

Authors since Foster and Madsen have differed on the degree to which they felt the Greco-Roman humoral theory matched, supplanted or complemented indigenous health beliefs. Ortiz de Montellano claimed the Aztecs were receptive to European humoral theories because it mirrored their own system and there was a fusion of cultural beliefs around the hot/cold duality, but the duality of wet/dry was rejected [18]. Other authors, such as Lopez Austin [20], concluded that indigenous hot/cold beliefs pre-dated Hispanic influences and persisted in spite of, and alongside, the introduction of outside influences.

The hot/cold classification process itself has at times been considered to be a “hard” classification; a particular food or type of food *is* always hot or cold, and differences were considered to be “discrepancies.” At other times, such discrepancies have been explained as resulting from a classification that is based not on the hotness or coldness of an element itself but on its successful use against an illness that is classified as hot or cold; i.e., a food that works to improve or re-balance a hot condition is, by definition, cold [9–13]. While some researchers discard the need for a full accounting [10], many others have concluded that varying contexts or domains of influence help determine classification. Mathews [36], for example, describes a complex system present in one community in Oaxaca, Mexico in which food items were classified in three ways: for their possible “dangerous” role in meal preparation, their ability to neutralize or moderate sexual behavior, and for their healthfulness in the treatment of illness. She recorded how certain items might be subject to classification as hot or cold in terms of their possible toxicity as a foodstuff, but have no classification in the dimensions of neutralization of sexual behavior, or healthfulness. She also documented how items can possess opposite classifications concurrently. White beans, for example are “considered to be ‘hot’ with respect to the ‘food danger’ dimension” and is therefore served with cold foods, but are ‘cold’ in terms of neutralizing ‘hot’ sexual states.” ([36], p. 838). Mathews’ approach emphasizes the need to understand belief and action in context and to not search for invariant classifications.

In summary, with regard to local health practices in the Americas, disagreements abound with respect to how much empirical evidence, sensory evidence, abstract symbolism, or any combination thereof are incorporated in hot/cold medical theory and practice. In addition, authors have disagreed with regard to how hot/cold beliefs are conceptualized and applied, or how rigidly systematized these are or even need to be [37–39]. Further, the degree of overlap with biomedical categorizations of disease or dis-order are imperfect: not every indigenous malady has an equivalent in the biomedical system of thought, and not every biomedically-defined disease is recognized within any local humoral system. Moreover, while it is generally accepted that indigenous healers and practitioners have a larger, more complex, more systematized system of knowledge than lay people in their communities, not every healer holds the same beliefs to the same degree [38]. While the basic conceptual apparatus and practice is recognizably and similar, the implementation of these differs somewhat from healer to healer and one circumstance to another. As Messer stated, “Even if they accept different traditional classifications for individual items or follow different paths to classification, they [humoral systems] still share a basic set of rules for classification, and faith in a common system” ([11], p. 139).

## Methods

Data reported here come from a 2012 qualitative study exploring domestic salt use in a Latino farmworker community in Fresno County, California. The study set out to examine the acceptability of salt fluoridation as a caries (dental decay) prevention strategy [40] in this Latino community that lacks fluoridated water [41] and has a disproportionately high prevalence of oral health issues [42–44]. Interviews and focus group discussions ranged widely, quickly and consistently yielding commentary on a very wide range of germane topics relevant to understanding salt use and its connection to health. Strong emergent themes in the data identified the importance of balance in health, and beliefs about the effects on health of an excess or lack of salt. These ideas are suggestive of humoral concepts and practices [9–13]. We report the ways in which ideas and actions based on humoral theory were invoked to explain illness causation and the role of salt in the prevention and amelioration of illness.

### Study site

California is a major agricultural state with high proportions of Latino migrant and non-migrant farmworkers. This qualitative research study took place in Mendota, Fresno County, a Latino farmworker community in California’s Central Valley (see map [45]). The community’s permanent resident population of 11,148 in 2010–2013, the period during which data were collected, was 97% Latino-origin, comprising primarily first-generation, but also some second-generation, immigrants from Mexico and other Latin American countries [46]. Non-livestock agriculture is the main economic enterprise, especially the growing of cantaloupes and tomatoes, as well as rice, fruits and other vegetables. Approximately 46% of the population lives in households with incomes at or below the United States (US) federal poverty level, defined in 2012 as an annual income less than or equal to $23,492 for a family of four [47, 48]. This high level of poverty is partly due to high seasonal unemployment, the continuing long-term impact of the nationwide economic downturn in 2007–2008, as well as the current drought [49].

People in this farmworker community, like migrant farmworkers generally, experience a range of chronic illnesses. In addition to cardiovascular disease and respiratory disease, these include diabetes, dermatological conditions, high blood pressure, anemia, poor dentition and oral health [42–44, 50–53]. Some of these conditions are directly associated with agricultural occupations and some are exacerbated by excess dietary consumption of salt. Environmental exposures (e.g., to pesticides) and climate extremes, especially excess heat, exacerbate many of the illnesses to which members of this population are prone [54]. During the summer and harvest times, daytime temperatures in this region regularly approach 100 °F. Heat waves, which can last for several weeks, at times bring temperatures exceeding 110 °F [55–57]. Under these conditions, minimizing fluid and sodium losses helps to prevent heat-associated illness for field workers by maintaining thermoregulation [58]. Significant impacts of excess heat can be seen for several disease categories, including cardiovascular disease, respiratory disease, dehydration, acute renal failure, heat illness, and mental health [55]. Thus far the literature contains little documentation of the practices in which Latino fieldworkers engage to establish rehydration, or the ways in which humoral concepts play a role in this.

### Procedures

Study procedures were approved by the University of California San Francisco’s Institutional Review Board (#10-04246), and conformed to the funder’s approved protocol and Clinical Terms of Award. In addition, all study procedures were informed by and approved by two local community advisory boards. Ethnographic mapping and observations of the community’s access to and use of salt along with interviews with key informants (such as restaurant and store managers, local physicians, civic leaders) occurred first. The study aimed to interview 25 to 30 participants and conduct five confirmatory focus groups with 5–8 participants in each group. In-depth interviews in Spanish with individuals in early 2012 were followed by confirmatory focus groups with individuals who had not previously been interviewed, in November 2012. Inclusion criteria for participants were: self-identification as an adult of Latino origin providing care to a child under age 10, and affiliated in some way with farm work (either as a field or crop worker, ranch hand, spouse of a farmworker, or employed in an allied industry such as produce packing or distribution). Participants were recruited through various child-related and community organizations. Each caregiver received $30 compensation for his or her time in the form of a gift card for food at a locally owned supermarket. Study procedures are also described more fully elsewhere [40].

We explored salt beliefs, knowledge, habits and use at home in cooking and eating, and at work while in the fields. In both interviews and focus groups we asked about concerns about salt consumption and its possible effects on health, especially but not exclusively its negative effects. Interviews and focus groups lasted 1.5–2 h with individual interviews conducted in participants’ homes and focus groups in a local community center. Each individual interview and focus group was audio recorded, translated into English, transcribed and imported into NVivo 10.0® qualitative software program [59]. The broadly social constructivist theoretical approach underpinning this study [60] relies on data collection and constant comparison of themes presented by participants to develop a conceptual model of participants’ ideas in relation to: (a) established views (called *a priori* codes derived from the literature) and (b) viewpoints emerging directly from the observational and text data (also known as *post* codes) [61–63]. Three study researchers independently and iteratively read the transcripts, identified and applied codes, analyzed and connected the emerging broad themes in the data. The first round of coding included six categories of classification: salt, work, health problems, food, children and fluoride. Subsequent more detailed analyses focused on the first three of these large categories and their overlaps, and were primarily conducted by the second author. A refinement of the initial salt code, for example, included differentiation of the term ‘sodium’ and ‘salt’, ‘table salt’ from salt used for other purposes such as in water softening devices. Overlaps between the code “table salt” and “work”, for example revealed the ways and reasons why salt featured in certain work settings. In this fashion, we used coding and interpretive practices similar to those described by Strauss and Corbin, especially when identifying emergent themes [61]. Confirmatory readings and discussion of the coding refinements and main themes/findings then occurred among the authors until a consensus was reached on data interpretation.

Illustrative quotations from unique individuals are included in the Results section. These comments are typical of the entire body of general comments on specific topics or ideas made by respondents.

## Results

### Study participants

Table 1 outlines the socio-demographic characteristics of participants. A total of 30 people engaged in individual interviews with another 31 participating in the five focus groups. This sample matched well the population targeted for inclusion. The majority of participants were women from Mexico and Central America. The majority of participants or their partners were involved in farm work, a proportion similar to that reported by Stoecklin-Marois and colleagues in a representative sample of the same community [46]. Our participants described their work as taking place in the cotton, fruit and vegetable fields, harvesting cantaloupe, watermelon, tomato, asparagus, onion, garlic, lettuce and broccoli. Major tasks entailed hoeing, pruning, cleaning, picking, sorting and packing in the produce packing plants and distribution centers or at the tomato cannery. All 61 participants were adults who self-identified as Latino with a low-income, with many also reporting a low educational attainment, the mean educational level of the sample being 6th grade (with a standard deviation of 4 grades). The level of acculturation for this community has been reported as “low” [46, 64].

**Table 1:** Socio-demographic Description of Participants (*N* = 61)

Participant Demographics	% (*n*) or Mean ± SD
Gender (female)	87% (53)
Age, in years	41 ± 12
Education (grade completed)	6 ± 4
Nativity	
• Mexico	57% (35)
• Central America	34% (21)
• US	8% (5)
Years living in US^a^	17 ± 9
Number of children in family	2 ± 1.3
Self or partner involved in farm work	84% (51)

^a^If foreign born

### Imbalance from exposure to extremes

Explanations of the causes of certain illnesses and efforts to prevent illness and restore health were offered by study participants through stories of their daily lives and through detailed descriptions of the actions they took as individuals and as a group when working in the fields. They told, for example, of sharing lemon, salt, and self-prepared liquid solutions to address issues such as extreme heat that threaten the hot-cold equilibrium of the healthy body. One study participant understood her asthma to be a result of constant exposure to extreme and sudden changes in temperature, going from a very hot environment inside of the tomato cannery to very cold air outside, an explanation consistent with biomedical views.

Study Participant: Look, during winter I went in [the factory] and when I came out it was cold and raining [makes troubled breathing sounds.]

*Interviewer: That you couldn’t breathe?*

Study Participant: Choking because I was coming from the heat to the cold, and that was the hardest.

She also attributed her asthma to inhaling hot steam from bags of tomatoes at the factory while they were being processed at high temperature.

Study Participant: I came out sick with asthma from there. I didn’t have asthma before but since you have to cut the bags [of cooked tomatoes]. The [wheelbarrow] they give you is very hot and you have to cut them really fast… cut it, and shake it and we burn our hands even with the gloves from how hot it is and you inhale all of the steam. That’s why there are a lot of sick people. Because you inhale all of the vapors [steam] from the bag.

For several study participants, colds and coughs common in children, were attributed not only to exposure to extreme weather during the summer and winter months, but also to wide or rapid oscillation in temperatures during the day and night. Here, temperature was meant in both a physical as well as a symbolic sense. One grandmother explained how colds and coughs develop by telling a story about her grandchildren:

Study Participant: Well, because sometimes we don’t pay enough attention and they [children] don’t take care of themselves. They take their clothes off when they’re sweating and that’s really bad, or they take off their shoes if you’re not watching them; they get wet.

Extreme exposures to elements in the environment (weather conditions, hot steam from the tomato cannery, pesticides in the soil, and pesticide “contaminated wind”), and situations in which the individual has limited or a complete lack of control over their work, were often described as issues of “too much” or “too little” (too much heat, too much stress, too much labor insecurity, too much worrying… too little rest, too little time to eat, too little money). Rapid changes in conditions such as these ‘shock’ the body and cause illness. Repeated or sustained exposures to these excesses and extremes also had implications for health. Participants talked about many instances in which prolonged excess of, or lack of, one element would provoke the body to become unbalanced, resulting in illness. One woman in our study identified arthritis as a common problem in this population, while another attributed hand pain to her hands being too frequently immersed in cold water or exposed to icy conditions:

Study Participant: … because the older you get, the more pains you get in your hand…I’ve packed the broccoli in mid-winter so much that by lifting the broccoli container you could see the ice underneath.

Participants recognized cardio-vascular disease or hypertension (high blood pressure) as a common health issue in the community (see also [65]). It is, simultaneously, a formally defined medical condition, diagnosed by physicians and treated biomedically and a ‘folk’ illness amenable to other explanatory schema and therapeutics. Members of the community added other symptoms and causes to this category of illness, in effect redefining what hypertension means: it became “*high blood pressure*”. This redefined condition was attributed to an accumulation of deep sadness, too much tension, too many worries, thinking too much about problems and working too much (especially in the fields), or resting too little. All of these feelings and exposures added up, accumulated in the body, and caused “*high blood pressure*.” In a recent study by Aroian and colleagues looking at hypertension prevention and beliefs among Latinos, participants identified certain emotions, such as feeling too “stressed out,” “excited,” “worried” or “upset,” as causes of hypertension [66]. The commonality uniting these emotions is that for the study’s participants they were classed as “hot,” a term that expressed a strong reaction.

In our farmworker community, hypertension as “*high blood pressure*” was seen as being an endemic problem because of the extremes and persistence of stress. “*High blood pressure*” was attributed to a persistent lack of food and water when needed; not eating enough or at normal times due to too many hours working in the fields (or, alternately, not having enough work due to the drought); having to wait for the foreman to announce a break, and having limited time to eat on breaks. According to one participant: “*as you can see, there is no work. They [people in the town] start thinking about the rent coming up. If they don’t have money to pay for it, then they’ll get kicked out to the street. All of that is expressed by people too.”*

When asked who suffers more from *high blood pressure*, one of our study participants said that it was women more than the men “*…because we [women] go here and there. When we come home from work we still have to cook, to wash and … well, the pressure we have. Like we have to go to work the next day, we have to make lunch, to get the things for the kids ready.*”

When situations were out of one’s control, and for situations in which distress was constant, it was difficult for the body by itself to restore balance and return to its normal state. In such instances, symptoms can be treated but often balance is restored only temporarily. Symptoms identified as being a result of “*high blood pressure*” were treated by actions such as talking to a neighbor, or someone one trusted, −“*to tell her things, to get it off of your chest”-* or going for a walk to distract oneself. Consistent with humoral healing practices, foods with particular characteristics were often used to alleviate symptoms of “*high blood pressure*” and other health issues. In order to restore balance resulting from “*high blood pressure*” or blood pressure oscillating from high to low, several participants used “dark soda” (e.g., Coca Cola® or Pepsi®) and a “pinch of salt.” Other therapeutic variations included combining dark soda, lemon and salt, or combining coffee with salt. The physiological symptoms of “*low blood pressure*,” as expressed by study participants, were low physical energy, vertigo, sweating and vomiting, often occurring in situations of sustained physical exercise and sweating, especially in extreme heat [67]. When referring to “*low blood pressure*,” one woman said she would also use a “red popsicle” in order to get her “energy to go up.” Madsen, working among the Tecospa in Mexico, noted that dark colors, including black, brown, dark green, purple, and red, were considered hot, with black and red the most important colors for classifying an object’s symbolic temperature [10]. Thus “hot” dark soda, coffee or a red popsicle would counteract the literal and metaphoric “cold” induced by sweating or water leaving the body. Several participants reported combining salt and coffee to prevent or address heat-related symptoms. Foremen would also provide salt and coffee to fieldworkers.

Study Participant: So, they [the foremen] would sometimes give us coffee with salt. According to them [foremen] so that we would feel better. The foreman would sometimes carry coffee. “Oh, I feel dizzy from the heat!” Wait, just wait, let me give you some coffee and salt.

*Interviewer: And they would give you that?*

Study Participant: They would give us that instead of sugar. You would feel better with that, I’m not going to say we didn’t.

Like “*high*” and “*low blood pressure*,” the development of diabetes was also attributed to states that were extreme, such as a persistent state described as ‘hunger,’ said to be caused by events similar to those inducing “*high blood pressure*”: lack of time to eat while working in the fields and foremen not giving workers a break. Work hours frequently extended from early morning to afternoon, such as 3:00 am to 1:00 pm, or 5:00 am to 4:00 pm, times not conducive to eating meals as a family. Stomach problems – abdominal pain, cramps – were attributed not just to excess heat but also to not eating well in the fields, a result of being too tired to eat, opting for rest during breaks instead of eating, and working too much.

### Salt in therapeutic practices

In general, preventing illness, addressing symptoms, restoring and maintaining health through re-establishing equilibrium were important daily goals for study participants who worked in the fields. Getting sick to the point of not being able to work was not an option. Work in the fruit orchards or vegetable fields lasted only part of the year and opportunities for a regular income outside harvest season were severely limited [68].

Not every medical condition recognized as prevalent in this community was subject to humoral explanation. Community members, for example, knew that, in common with many Latino farmworker communities nationwide they - and their children especially - had high rates of dental decay (caries) and unmet dental care needs [69–71]. Study participants were interested in, even eager to find caries preventive that was safe, effective, easy to use, and low cost. They rejected the usual approach, water fluoridation, for a variety of reasons, importantly the shared belief that the tap water was unsafe and unpleasant to drink. Residents in this community have reported that the city’s tap water commonly smells of sewage and chlorine, and has a yellow appearance particularly in the mornings [41]. Participants expressed a willingness to embrace an effective alternate caries preventive if it were easily available– namely, salt fluoridation [40]. At no point, however, were concepts derived from humoral pathology, diagnostics, treatment or practice invoked in their discussions about caries, its prevention, recognition or treatment.

#### Salt as beneficial: restoring balance

Salt was one of the most frequent food items participants mentioned for preventing illness and restoring health. In addition to the use of salt at home and in the fields to treat dehydration in children and adults, salt was used in a number of other healing practices, too: to treat stomach aches or indigestion, fever blisters, pain, to bring down swelling, for mosquito bites, and to relax the feet when soaking them in water.

*Interviewer: So you don’t use it [salt] for anything else?*

Study Participant: When [the children] have a [insect] bite, I grab a pinch of salt and I put it there, for everything.

*Interviewer: Like for…?*

Study Participant: To heal.

Other than the work itself, no more individual and collective effort was expended in the fields than those directed toward avoiding dehydration and managing symptoms of heat-related illnesses. To replenish the loss of water while working in the fields, often under extreme heat conditions, participants were cognizant of the need to drink water, but not too much water. Noted one participant:

Study Participant: It was like one hundred and fifteen degrees and we did start to get dehydrated. We were very thirsty and we kept drinking water until our stomachs swelled up from drinking so much water and I started to vomit it up through my nose and mouth…

Another participant commented:

And what I do in order not to [get sick] is take a little bag with lemonade [dried lemon and salt packet]. I come and I add a little bit to the container that I use to drink water and that’s what I drink. I mix natural water with that, because if I drink the water just like that, that’s it, I can’t work anymore, because it’s bad for me…

In this California Central Valley community, fresh fruits and vegetables (watermelon, green apples, cucumber, tomatoes, avocados, melon, jicama, oranges and lemons) were eaten with salt and chili, or with *pico de gallo*, a common practice in Latin cuisines [72]. *Pico de gallo* is a prepared powder bought at the local stores consisting of salt, lemon, and (spicy) chili. Both children and adults consumed salt or *pico de gallo* with fruit at home and at work to “refresh” themselves in response to the effects of heat. One study participant stated that *“People who work in the field carry salt]. Because of the heat, the salt helps them… because the salt helps them to refresh when they sweat.”*

#### Salt as potentially dangerous: out of balance

Although ingesting salt was discussed as an essential survival tool for working in the fields, study participants also approached salt with caution. The biomedical critique of excess salt for its deleterious cardiovascular health effects on the body was common knowledge and widely accepted, a health concern people expressed. Many people had been exposed to this information from doctors or through health education efforts. Participants, their family members or other members of the community had at various times been counseled to reduce salt consumption to prevent or address hypertension or the impact of diabetes. However, since salt affects food taste and texture [7], many people struggled with reducing their daily salt intake. In their view, under-salted food lacked flavor. It tasted “flat,” “bland,” or “plain.”

Under-salted food could also lack (humoral) efficacy as it was also believed that too little or too much of a substance could have the opposite effect than the one desired and cause further harm. Too little salt could possibly not restore balance or stave off the ill effects of exposure to excess heat, disordered eating patterns and unrelenting everyday stresses. Excess salt could be dangerous, and not just for cardio-vascular health. Salt, then, was both a cause of illness and a therapeutic tool. It all depended on symptoms, body part affected, circumstances, diagnosis, and prior experiences [9].

A few study participants associated excess use of salt with possible damage to the kidneys and pancreas as well as to the liver. One participant described this as “*le pica uno el higado*” (“it [excess salt] pokes holes in [stings] your liver”). Despite reporting that his doctor had told him it was due to other causes, in our study a 40-year-old farm worker on dialysis associated his kidney illness, at least in part, to the excess use of salt growing up and then in the fields as an adult. The following discussion occurred during a focus group:

Study Participant 1: And since we were brought up with just salt… well, I’m from El Salvador, and we were raised on nothing but salt. We grew up with lots of salt. And, well… the doctor hasn’t told me what caused this illness, he said it’s bacteria, but maybe the salt also hurt me.

Study Participant 3: Yes, lots of salt.

*Interviewer: Mr. [A], when you say you were raised on nothing but salt, what do you mean by that?*

Study Participant1: That there was nothing but tortillas and sometimes there was just salt. My mom would put the salt on the table and sometimes that was all… because we didn’t have beans. There was nothing else.

Horton’s recent work has elaborated on other aspects of this belief associating salt, exposure to excess heat, physical and emotional stress, constant vigilance and worry to kidney and liver disease in this population of Latino farm workers [68].

## Discussion

Many ideas and practices long-associated with humoral medicine in Mexican and Central American contexts are present in this contemporary Latino farmworker community in California’s Central Valley. Our results show the persistence of the hot/cold humoral dichotomy in this Latino farmworker community in the US, and beliefs regarding the importance of balance in the maintenance of good health and in preventing the ill effects of extreme conditions. Our data also reveal how salt is used to treat symptoms of illness and to respond to severe heat exposure (see also [59]). Study participants used these ideas to explain the causes of, and appropriate responses to, certain health conditions and symptoms (cf. [25, 28, 29, 38, 39, 68]). These ideas appear to be related to health practices and disease/illness concepts that existed in pre-Conquest or Conquest eras in both Central and South America. A major focus is on the hot/cold axis with far less emphasis being placed on other aspects of humoral explanations (eg, ‘fresh’). Other researchers have occasionally noted a persistent commentary on the hot/cold dichotomy in relation to health/illness in their work with Latino populations in the US. For example, Lam and colleagues note ideas of hot/cold in relation to farm workers’ management of heat illness [73]. Thus far, however, ideas and practices around hot/cold have not been explicitly linked to nor seen as guided by an underlying systematic theory of medicine with respect to this topic.

In his description of the health beliefs of the people of the Mexican village Tzintzuntzan, Foster [9] noted that two types of heat and cold, one physical and the other metaphoric, can, by themselves or in combination, upset the equilibrium that maintains a healthy state. Both ambient temperature, such as a hot or cold day, and hot and cold attributes (*calidad*) believed to be a characteristic of foods, herbs and other substances, can affect one’s health equilibrium. He describes how the body is rarely in a stable state of equilibrium, but fluctuates in its state of physical and metaphoric hot and cold. This in itself does not constitute illness, but rather leaves a person vulnerable to illness. For example, exposure to perceived physically heating activities, such as eating, exercise or pregnancy, or exposure to the metaphoric heating qualities of certain foods and drinks, lead to an “‘above-normal’ state of heat” ([9], p. 809). Preventive medicine, therefore, involves reducing the risk of additional heat or cold exposure especially if these elements are applied too quickly or are too extreme. It is only when such fluctuations reach sustained extremes that illness can result and therapies must be applied to restore equilibrium [9].

While there have been claims that it is not usual for migrants to carry humoral ideas into the US from Latin America or the Spanish-speaking Caribbean (e.g., [74]), most commentators note the persistence–albeit often in a somewhat adapted form - of humoral concepts and practices in migrant settings [75–77]. We found that humoral medicine informs the Latino migrant community’s material and symbolic use of and meanings given to salt as a culinary and therapeutic agent. Data point to a robust hot/cold dichotomy and ideas of ameliorating excesses or imbalances. Our results are similar to other reported findings.

Just as we found for respiratory conditions like asthma, an early study of hot and cold beliefs in the Mexican village of Tlayacapan, Morelo, Ingham [17] reported that people in the village believed that respiratory illnesses were caused by *aires* (a cold draft). A person would be vulnerable to an *aire* when moving from a warm to a cold place. When heat was displaced upward by cold entering the body from the cold floor it could cause *calor subido* (risen heat) or high fever [17]. More recent work has also examined the role of alternate medical beliefs in recognizing and responding to asthma by Latino populations [73, 78–81]. Mitchell and co-authors [78] looked at families’ beliefs about causes of asthma and medical treatment among caregivers of children with asthma from Puerto Rico and the Dominican Republic. Some of their study participants lived on the island of Puerto Rico and some in Rhode Island in the US. Their questionnaire with 100 primary caregivers included a sub-category of humoral etiologies (e.g., exposure to hot and cold elements), and revealed that a higher proportion of island Puerto Rican caregivers than other caregivers believed that getting wet while sweating, bathing while sick, and very hot weather would cause asthma [78]. Pachter and colleagues also investigated asthma beliefs and practices among mainland Puerto Ricans, Mexican-Americans, Mexicans, and Guatemalans and noted that in addition to mainstream biomedical interpretations, aspects of the humoral (“hot/cold”) theory were expressed [79]. Echoing a participant in the study reported here, who complained about her grandchildren taking their shoes off and getting their feet wet as a cause of respiratory problems, Mexican and Guatemalan participants gave numerous hot/cold causes for asthma (such as ‘taking a bath while having a cold or flu,’ ‘drinking icy drinks when one is sweating,’ ‘walking on a cold floor without shoes,’ or ‘getting wet while sweating’). Connecticut, Mexican, and Guatemalan samples all reported that exposure to cold can cause asthma [79].

Puerto Rican study participants in Harwood’s study [16] believed arthritic pain in the hands came from putting hands in cold water after they have been in hot water, a concept similar to one expressed by another participant in our study. Given the prevalence of joint and muscle pain experienced by rural fieldworkers [50–53, 76, 82], it would seem humoral concepts would be widely invoked as both cause and therapy for this class of malady. In her 1959 ethnography of a migrant Mexican community in the US, Clark ([75], chapters 7 & 8) noted the central and extensive role that humoral concepts and medical practice played in life in the urban *barrio*. Specialist healers attended to specific types of disorder using plant-based dietary items to redress a wide variety of hot-cold imbalances.

A frequent topic throughout the humoral literature about Spanish-speaking peoples is the concept of what we have described as “*high blood pressure,*” or *presión.* Among a diverse group of Latino women (Puerto Rican, Mexican, Central and South American, and Cuban) residing in Orange County, Florida, researchers found that although participants were generally knowledgeable about the biomedical causes and risk factors associated with hypertension, they also had other explanations. Similar to our findings, Orange County participants associated hypertension with feeling “too stressed out,” “excited”, “worried” or “upset” [66]. These emotions, thought to be stronger among women than men, were an issue whether they were expressed openly or kept as private thoughts. Thus, both physiological and culturally-defined forms of high blood pressure explain the symptoms participants described. The material (physiological) and symbolic/ metaphoric (cultural) aspects of hypertension/“*high blood pressure*” co-exist and intertwine [15, 66, 75, 77].

Repeatedly across the decades in which Latin humoral systems of medicine have been examined “*high blood pressure*” has been attributed to unrelieved and irreducible stresses that disorder and fragment lives into patterns perceived to be different from the culturally normative or desirable. In a seminal paper, Hunt and co-workers [83] describe how particular circumstances come to be conceptualized as responsible for illness onset; they call these circumstances “provoking factors.” Prolonged or recurrent undesirable behaviors, such as frequent drinking of alcohol to excess or marital discord, can provoke imbalance and illness. Emotional upheavals, resulting in people “thinking too much” or “worrying a lot”, are also provoking factors. Fright and anger (often preceded by nervousness or irritability) have long been described as major emotional states or provocations giving rise to so-called “folk illnesses” widely known throughout Mexico as *susto* and *bilis,* respectively ([75], pp. 162–217). A variety of therapies, traditional, humoral and biomedical, singly or in commination, are applied to provoked illnesses. Though we discerned hints of a gendered nature of recognition of or response to various types of provoking factor, this is not yet a topic extensively elaborated in the literature.

People search for explanations for their out-of-control lives and chronic illnesses. They examine their biographies to locate the source and nature of provocations. Sufferers construct and re-construct their life stories as various provocations arise or abate and as they seek to ameliorate disorder. Describing this process as “living in the subjunctive,” Good and colleagues [84] argue that such biographical narratives have the power and potential to heal precisely because they present multiple perspectives and suggest alternative plots and variable time frames about the source and possible outcomes of illness. Even though constantly shifting to some degree, subjunctive life narratives justify continued care-seeking and maintain hope for positive outcomes as provocations come and go or change in nature. “*High blood pressure”* is a phrase that encapsulates living in the subjunctive for these Latino farmworkers.

Unrelenting physical stress and trauma (eg, extreme heat or work-related injury) are major provoking factors. We concur with Horton [68] and include in the notion of provoking factors macro-level structural factors. She shows how structural level inequities, arising for example from federal migration policies, unequal application of labor laws, poor enforcement of occupational safety regulations and varying economic incentives (piecework versus hourly wage), lead to unrelenting stresses for fieldworkers. And a collective sense that these cumulative and sustained stresses are a major cause of disease, particularly but not only kidney failure, excess morbidity and premature death. While amelioration of these structural issues is beyond individual workers, the impact of these intertwined phenomena is not beyond their comprehension. Migrant farmworkers understand clearly how the multiple contexts of their lives intersect ([68], chapters 1 & 2; [84–86]), how structural vulnerabilities influence their behaviors and thoughts, even if their discourse about the impact of these provoking factors is largely vested in accounts of individual actions in daily life.

Each community has its own local construction, deployment and management of ideas about the causes and treatment of illness, be those ideas based on humoral or other systems. There is no necessary one-to-one correspondence between disorders recognized, discussed or treated in the humoral or biomedical system. A detailed account from San Miguel Tulancingo, Oaxaca, Mexico reveals the hot-cold system to be complex, often indirectly applied and not comprehensive in its coverage [87]. While some aspects of particular hunoral vulnerabilities or conditions may resonate partially with descriptions of ailments described in other explanatory schema, some disorders appear to fall completely outside the purview of the humoral approach. For example, caries (dental decay) apparently does not feature in humoral understandings while other humorally-defined infirmities are unrecognized in biomedicine (eg., ‘fright’ disorder. ‘*susto’*, or ‘*mal de ojo’*) [13, 17, 75, 87]. In San Miguel Tulancingo, musculo-skeletal disorders are virtually always subject to humoral explanation and treatment whereas injury is almost never conceptualized in this fashion [87]. Many other categories of disorder have mixed explanations depending on circumstances and presentation of symptoms. Nor is it necessarily the case that traditional or humoral therapies will supplant biomedical therapies, even when used simultaneously. Herbal potions may accompany but be a minor aspect of a therapeutic regimen, as demonstrated clearly by Latinos with diabetes on insulin therapy [88].

While it is not possible to simply extrapolate specific practices from one community to another location or local system of thought and practice, we must remember Messer’s important commentary on the *logic* of humoral systems. She reminds us, “Even if they accept different traditional classifications for individual items or follow different paths to classification, they [humoral systems] still share a basic set of rules for classification, and faith in a common system” ([11], p. 139). This caveat is eloquently further detailed in a recent paper by Garcia-Hernandez and colleagues [87].

Despite their paucity, there are a few intriguing prior reports on salt specifically as a therapeutic humoral agent. Foster ([12], p. 102) compared his work in Tzintzuntzan with Mathew’s examination of humoral beliefs in Oaxaca, Mexico [36]. He points out that while there is a high level of agreement within each community with respect to the humoral value assigned to salt (100% of Mathew’s sample, and 91% of Foster’s sample), in Oaxaca salt is considered cold whereas in Tzintzuntzan it is considered hot [12]. Hence, humoral systems of thought in relation to health and illness are very much local systems that operate within circumscribed geographic regions or cultural groups, produced by interacting sets of people with shared beliefs in the fundamental underlying principles of symbolic and material balance of properties. While people from different groups would follow the basic principles guiding any humoral system, they would learn the precise associations made by their particular group. Nevertheless, despite decades of work and some substantial disagreements in these areas, there is near-universal agreement that hot/cold principles and classifications have been, and still are, utilized in some Latin American communities for diagnostics, health prevention, health maintenance and therapy [89].

Our results show that in addition to plant-based medicaments, other natural substances such as salt can be centrally incorporated into humoral healing systems. However, the extent to which non-plant substances are systematically used in humoral medicine is at present largely unknown. One report [90] describes low-income Mexican-origin women in Southern California in the US consuming clay during pregnancy. Whether this practice – known as geophagia or pica - was informed by humoral concepts is unknown; nor do we know the extent to which clay may contain salt or other salty substances. Clay ingestion has been claimed to counteract diarrhea or address anemia or other illnesses resulting from poor nutrition [91, 92]. While the prolonged ingestion of clay and other non-nutritive materials (eg, dirt, ice, paint, hair) is currently classed biomedically as dangerous, possibly leading to physical or developmental disability in children or connected to medical and psychiatric issues in women [91–93], the craving and intermittent consumption of clay, especially during pregnancy, has been documented for centuries in many different regions worldwide.

Authors have consistently reported practices other than ingestion of plant medicaments also being used to treat humoral imbalances. Thus, the therapeutic armamentarium of humoral systems is broad and complex. Among other common therapies are baths and sweat lodges (*baños*), cleansings or purifications (*limpia*), cuppings, application of poultices, and retreat from social interaction for specified periods or in certain circumstances [38, 67, 68]. A variety of specialist therapists exist, often known as *curanderos* or herbalists as well as *sobadores* who deal predominantly with musculo-skeletal problems [74, 75, 82, 87]. Though often mentioned in various reports as empirical findings, how the bodily practices of bathing, purification practices, and so forth, cognitively or practically mesh with therapies based on the consumption of various materials, such as herbs, is not yet well detailed in the literature. Nor is it always clear what type of local healer directs the application of which therapeutic actions. The role of salt in these adjuvant healing practices, particularly those aimed at preventing illness, is largely unexamined.

### Limitations

This qualitative study is limited because it involves a small convenience sample of low-income, Latino individuals in a single, rural site. Caution must therefore be exercised in generalizing results, especially to other Latino groups with different socio-economic backgrounds, migration experiences, greater health literacy or who reside in other geographic areas in the US. Nevertheless, data from our California Central Valley community are robust and consistent with findings from other contemporary studies with Spanish-speaking Latino groups, in both the US and elsewhere. These studies suggest that influences from humoral theory are likely to be found among (rural) migrant Latino groups, and therefore merit continued study. Investigation is needed into the ways Latino farmworkers conceive vulnerability to illnesses, both biomedical and humoral, and what they do to combat, ameliorate or prevent those ailments. The influence of humoral beliefs on rural field and factory workers’ management of occupational exposures, work endurance and health, particularly in conditions of extreme heat are especially worthy of further detailed ethnographic investigation. While empirical observations of the existence, expressions and processes of illness and illness prevention often match the perspective of a contemporary biomedical model, a Latino fieldworker’s understanding of the underlying causes of these expressions and processes can differ dramatically from those of biomedically trained personnel.

## Conclusion

Many of the ideas and practices associated with Mexican and Central American humoral medicine are present in this contemporary Latino farmworker community in rural California, and inform their material and symbolic use of and meanings given to salt as a culinary and therapeutic agent. Some of these ideas appear to be centuries old, existing prior to the Spanish conquest or arising shortly thereafter. Data point to a robust hot/cold dichotomy pertinent to many common illnesses experienced in Latino farmworker communities, to beliefs around the deleterious health effects of extreme conditions, the importance of balance in the maintenance of good health, and finally to the use of salt to treat symptoms of illness, especially but not exclusively those resulting from extreme heat exposure.

Humoral ideas guide people to recognize, interpret, understand and respond to symptoms and signs of illness and stress, to feelings of being out of control and being unable to manage life or stressful events. Humoral theory provides a useful framework for understanding how this population explains and attempts to prevent and ameliorate certain health conditions, providing an alternate worldview of illness and healing that biomedical practitioners need to be aware of—both in terms of understanding symptom description/presentation, and in making health education relate better to patients.

## Abbreviations

NIDCR: National Institute of Dental and Craniofacial Research
US: United States

## Footnotes

1 The Mayans occupied an area of around 325,000 square kilometers in southern Mexico and northern Central America. The Mayan civilization had three periods: the Formative Pre-classic (2,000 B.C–A.D. 250), the Classic (A.D. 250–900) and the Post-classic (A.D. 900–1519) [21]. The Late Classic to post-classic (colonial) period, about 300–1500 Century A.D. is being referenced here.

2 The Aztec Empire was located in central Mexico, which includes the Valley of Mexico. The Late Aztec period was A.D. 1350–1520, just prior to the Spanish Conquest [22].

3 Mesoamerica extends from central Mexico down through Central America, including Guatemala, Belize, Honduras and El Salvador. In addition to the Mayans and Aztecs, many other civilizations emerged in Mesoamerica between 1500 B.C. and the arrival of the Spaniards in the early 1500s A.D.
